# Supplementary material for: Kinetics of Moisture Loss and Oil Absorption of Pork Rinds during Deep-Fat, Microwave-Assisted and Vacuum Frying
Source: Foods. 2021 Dec 6;10(12):3025. doi: 10.3390/foods10123025 (PMC8701915; doi:10.3390/foods10123025)
Supplement: Supplementary file 1 [file foods-10-03025-s001.zip › foods-1464674 - supplementary - r2.pdf]

Supplementary files

Table S1a. Correlation coefficients between traditional frying time of pork rind and various experimental results.

a	Water content	Water activity	Breaking force	Oil content	Puffing ratio	CIE L*	CIE a*	CIE b*	CIE △E
Water content	1								
Water activity	0.956**	1							
Breaking force	0.691**	0.723**	1						
Oil content	-0.942**	-0.953**	-0.648**	1					
Puffing ratio	-0.880**	-0.902**	-0.752**	0.897**	1				
CIE L*	-0.706**	-0.782**	-0.605**	0.762**	0.701**	1			
CIE a*	0.467*	-0.549**	0.375	-0.480*	-0.465*	-0.778**	1		
CIE b*	-0.229	-0.200	0.041	0.335	0.203	0.056	0.355	1	
CIE △E	-0.741**	-0.799**	-0.581**	0.788**	0.700**	0.987**	-0.771**	0.043	1

* indicate significance at $p<0.05$; ** indicate significance at $p<0.01$. Not include t = 0 in statistical analysis.

Table S1b. Correlation coefficients between traditional frying time of pork rind and various experimental results.

b	Water content	Water activity	Breaking force	Oil content	Puffing ratio	CIE L*	CIE a*	CIE b*	CIE △E
Water content	1								
Water activity	0.929**	1							
Breaking force	0.691**	0.723**	1						
Oil content	-0.957**	-0.852**	-0.648**	1					
Puffing ratio	-0.880**	-0.902**	-0.752**	0.705**	1				
CIE L*	-0.897**	-0.791**	-0.605**	0.938**	0.701**	1			
CIE a*	0.818**	-0.701**	0.375	-0.857*	-0.465*	-0.944**	1		
CIE b*	-0.363	-0.319	0.041	0.397	0.203	0.295	-0.102	1	
CIE △E	-0.741**	-0.799**	-0.581**	0.788**	0.700**	0.987**	-0.771**	0.043	1

* indicate significance at $p<0.05$; ** indicate significance at $p<0.01$.

Include t = 0 in statistical analysis.

Table S2a. Correlation coefficients between microwave frying time of pork rind and various experimental results.

a	Water content	Water activity	Breaking force	Oil content	Puffing ratio	CIE L*	CIE a*	CIE b*	CIE △E
Water content	1								
Water activity	0.952**	1							

Breaking force	0.897**	0.901**	1						
Oil content	-0.951**	-0.897**	-0.876**	1					
Puffing ratio	-0.941**	-0.950**	-0.925**	0.913**	1				
CIE L*	-0.929**	-0.884**	-0.842**	0.929**	0.916**	1			
CIE a*	0.352	0.254	0.171	-0.397	-0.355	-0.475*	1		
CIE b*	-0.535*	-0.503*	-0.417	0.457*	0.333	0.393	0.357	1	
CIE △E	-0.943**	-0.892**	-0.838**	0.932**	0.898**	0.990**	-0.400	0.514*	1

* indicate significance at $p<0.05$; ** indicate significance at $p<0.01$. Not include t = 0 in statistical analysis.

Table S2b. Correlation coefficients between microwave frying time of pork rind and various experimental results.

b	Water content	Water activity	Breaking force	Oil content	Puffing ratio	CIE L*	CIE a*	CIE b*	CIE △E
Water content	1								
Water activity	0.969**	1							

Breaking force	0.897**	0.901**	1						
Oil content	-0.897**	-0.923**	-0.876**	1					
Puffing ratio	-0.941**	-0.950**	-0.925**	0.913**	1				
CIE L*	-0.977**	-0.924**	-0.842**	0.835**	0.916**	1			
CIE a*	0.840**	0.739**	0.171	-0.649**	-0.355	-0.898**	1		
CIE b*	-0.878**	-0.820*	-0.417	0.694**	0.333	0.876**	-0.704**	1	
CIE △E	-0.943**	-0.892**	-0.838**	0.932**	0.898**	0.990**	-0.400	0.514*	1

* indicate significance at $p<0.05$; ** indicate significance at $p<0.01$.

Include $t = 0$ in statistical analysis.

Table S3a. Correlation coefficients between vacuum frying time of pork rind and various experimental results.

a	Water content	Water activity	Breaking force	Oil content	Puffing ratio	CIE L*	CIE a*	CIE b*	CIE △E
Water content	1								
Water activity	0.952**	1							
Breaking force	0.857**	-0.904**	1						
Oil content	-0.769**	-0.745**	-0.703**	1					
Puffing ratio	-0.976**	-0.981**	-0.901**	0.787**	1				
CIE L*	-0.867**	-0.875**	-0.821**	0.744**	0.862**	1			
CIE a*	0.652**	0.604**	0.543*	-0.633**	-0.610**	-0.707**	1		

CIE b*	-0.820**	-0.826**	-0.794**	0.588*	0.802**	0.893**	-0.448	1	
CIE △E	-0.880**	-0.886**	-0.833**	0.732**	0.873**	0.997**	-0.687**	0.920**	1

* indicate significance at p<0.05; ** indicate significance at p<0.01.
Not include t = 0 in statistical analysis.

Table S3b. Correlation coefficients between vacuum frying time of pork rind and various experimental results.

b	Water content	Water activity	Breaking force	Oil content	Puffing ratio	CIE L*	CIE a*	CIE b*	CIE △E
Water content	1								
Water activity	0.941**	1							
Breaking force	0.857**	-0.904**	1						
Oil content	-0.902**	-0.819**	-0.703**	1					
Puffing ratio	-0.976**	-0.981**	-0.901**	0.787**	1				
CIE L*	-0.962**	-0.905**	-0.821**	0.870**	0.862**	1			
CIE a*	0.873**	0.769**	0.543*	-0.759**	-0.610**	-0.905**	1		
CIE b*	-0.849**	-0.877**	-0.794**	0.706**	0.802**	0.882**	-0.700**	1	
CIE △E	-0.880**	-0.886**	-0.833**	0.732**	0.873**	0.997**	-0.687**	0.920**	1

indicate significance at p<0.05; ** indicate significance at p<0.01.

Include t = 0 in statistical analysis.

Table S4 The correlation coefficient between the vacuum frying time and the test results.

a	Frying time	Water content	Oil content	Breaking force	Water activity	The puffing ratio
Frying time	1					
Water content	-0.899**	1				
Oil content	-0.197	0.273	1			
Breaking force	0.462	-0.672**	-0.346	1		
Water activity	-0.939**	0.958**	0.343	-0.567**	1	
The puffing ratio	0.872**	-0.884**	-0.221	0.605**	-0.883**	1

b	Frying time	Water content	Oil content	Breaking force	Water activity	The puffing ratio
Frying time	1					
Water content	-0.751**	1				
Oil content	-0.480	-0.846**	1			
Breaking force	0.462	-0.672**	-0.346	1		

Water activity	-0.962**	0.759**	-0.433	-0.567**	1	
The puffing ratio	0.872**	-0.884**	-0.221	0.605**	-0.883**	1

** indicate significance at p<0.01.

(a) Not include t = 0 in statistical analysis
(b) Include t = 0 in statistical analysis

Dried pork skin:

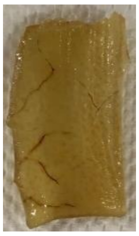

Traditional deep frying:

(0.5 min) (1 min) (1.5 min) (2 min) (3 min) (4 min) (5 min)

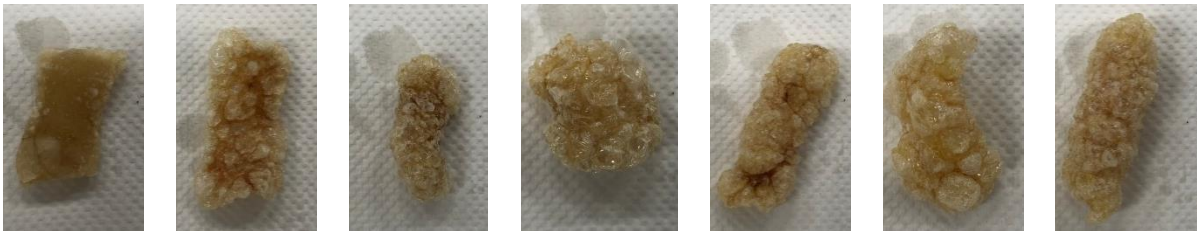

Microwave assisted frying:

(0.5 min) (1 min) (1.5 min) (2 min) (3 min) (4 min) (5 min)

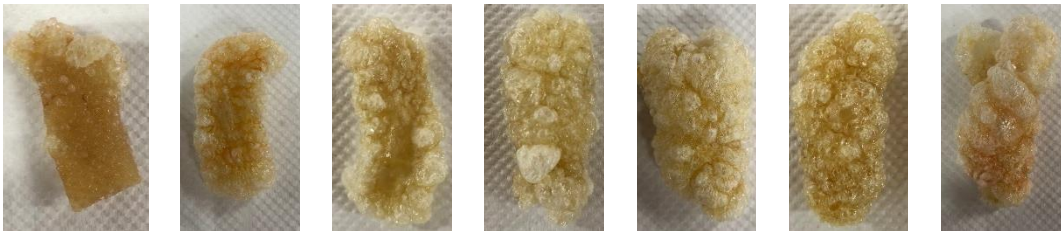

Vacuum frying:

(4 min) (8 min) (12 min) (16 min) (20 min) (24 min)

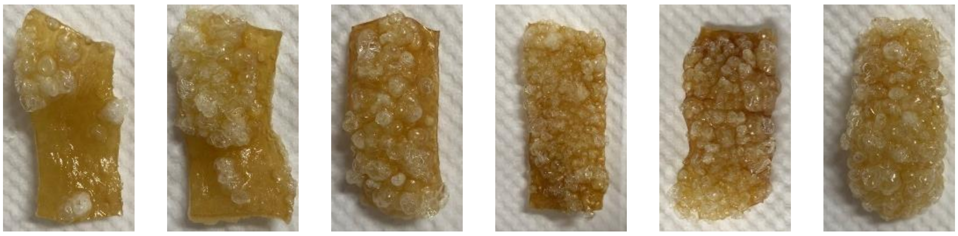

Figure S1 Appearance of fried pork rind by different frying methods and frying time.
